# A Review of Manganese(III) (Oxyhydr)Oxides Use in Advanced Oxidation Processes

**DOI:** 10.3390/molecules26195748

**Published:** 2021-09-22

**Authors:** Daqing Jia, Khalil Hanna, Gilles Mailhot, Marcello Brigante

**Affiliations:** 1 Institut de Chimie de Clermont-Ferrand, Université Clermont Auvergne, CNRS, Clermont Auvergne INP SIGMA Clermont, F-63000 Clermont-Ferrand, France; daqing.jia@etu.uca.fr (D.J.); gilles.mailhot@uca.fr (G.M.); 2 École Nationale Supérieure de Chimie de Rennes, Université Rennes, CNRS, ISCR–UMR6226, F-35000 Rennes, France; khalil.hanna@ensc-rennes.fr; 3 Institut Universitaire de France (IUF), MESRI, 1 rue Descartes, 75231 Paris, France

**Keywords:** Mn(III) (oxyhydr)oxides, water treatment, radicals, AOPs

## Abstract

The key role of trivalent manganese (Mn(III)) species in promoting sulfate radical-based advanced oxidation processes (SR-AOPs) has recently attracted increasing attention. This review provides a comprehensive summary of Mn(III) (oxyhydr)oxide-based catalysts used to activate peroxymonosulfate (PMS) and peroxydisulfate (PDS) in water. The crystal structures of different Mn(III) (oxyhydr)oxides (such as α-Mn_2_O_3_, γ-MnOOH, and Mn_3_O_4_) are first introduced. Then the impact of the catalyst structure and composition on the activation mechanisms are discussed, as well as the effects of solution pH and inorganic ions. In the Mn(III) (oxyhydr)oxide activated SR-AOPs systems, the activation mechanisms of PMS and PDS are different. For example, both radical (such as sulfate and hydroxyl radical) and non-radical (singlet oxygen) were generated by Mn(III) (oxyhydr)oxide activated PMS. In comparison, the activation of PDS by α-Mn_2_O_3_ and γ-MnOOH preferred to form the singlet oxygen and catalyst surface activated complex to remove the organic pollutants. Finally, research gaps are discussed to suggest future directions in context of applying radical-based advanced oxidation in wastewater treatment processes.

## 1. Introduction

Over the past few decades, with the rapid development of industrialization and the increase of anthropogenic activities, huge amounts of organic and inorganic contaminants were discharged into the surface and ground waters, causing water pollution problems and threatening human health [1,2,3]. However, conventional water treatment technologies, such as filtration [4,5], precipitation [6,7], coagulation–flocculation [8,9,10], and biological treatment [11,12] exhibited a minimal effect on the removal of recalcitrant pollutants. Therefore, there is an increasing demand for efficient, economical, and environmental-friendly water treatment technologies. Advanced oxidation processes (AOPs) have attracted particular attention due to their high efficiency for removal of recalcitrant contaminant. AOPs are able to remove and mineralize most unbiodegradable pollutants into harmless compounds, such as CO_2_, $H_{2}O$, and inorganic ions [13]. Based on various reaction conditions, AOPs can be classified into different categories, including Fenton reaction [14], Fenton-like reaction [15,16], photochemical oxidation [17,18], ultrasonic oxidation [19,20], electrochemical oxidation [21,22], ozone oxidation [23,24], and sulfate radical-based AOPs (SR-AOPs) [25,26,27]. Among them, the application of SR-AOPs for the removal of stubborn pollutants has received increasing attention due to their advantages. For instance, sulfate radical (${\mathrm{SO}}_{4}^{•-}$) has a longer lifetime compared with the hydroxyl radical (${\mathrm{HO}}^{•}$), a wide range of pH adaptation, and a high reduction potential (2.5-3.1 V vs. NHE) [28]. 

Generally, the peroxydisulfate (PDS, $S_{2}O_{8}^{2-}$) and peroxymonosulfate anions (PMS, ${\mathrm{HSO}}_{5}^{-}$) are employed as the radical precursors for producing sulfate radicals through breaking the O-O bonds of precursors. In comparison with PMS, PDS has a longer O-O bonds distance (1.497 vs. 1.460 Å) and lower bond energy (140 vs. 140–213.3 kJ/mol) [29,30]. Therefore, PDS is theoretically easier than PMS to be cleaved to generate ${\mathrm{SO}}_{4}^{•-}$. However, considering the unsymmetrical structure of PMS, it was reported that PMS activation was convenient for the removal of organic pollutants [31,32]. There are various ways to activate PMS and PDS to produce ${\mathrm{SO}}_{4}^{•-}$, for example, heat, UV, alkaline solution, metal ions, and minerals [33,34,35,36,37].

The activation of PMS/PDS by different transition metal ions (i.e., Co(II), Ru(III), Fe(II), Fe(III), Ag(I), Mn(II), Ni(I), and V(III)) for organic pollutant degradation has been reported [32]. The results showed that PMS can be efficiently activated by Co(II) and Ru(III), while Ag(I) was identified as the best catalyst for PDS activation. However, the high price of Ag(I), Ru(III), and Co(II) restricts their application in practical water treatment. In comparison, the activation of PMS/PDS by the transition metal-based minerals (such as magnetite, birnessite, and manganite) has attracted much attention due to their various advantages, such as wide resources, easy recycling, and low energy requirement [38,39]. Among the transition metal oxides, the manganese oxides have been widely developed in PMS/PDS activation for recalcitrant pollutant degradation due to their excellent properties, such as various Mn valences, ubiquitous existence, cost-efficiency, and low toxicity [40]. For instance, Zhu et al. employed the β-MnO_2_ nanorods to activate PDS for the removal of phenol. Efficient degradation of phenol was achieved in β-MnO_2_/PDS system through the generation of singlet oxygen (^1^$O_{2}$) [41]. Zhou et al. indicated the higher catalytic property of α-MnO_2_ than δ-MnO_2_ in PMS activation for 4-nitrophenol degradation because α-MnO_2_ owns more active sites, larger Brunauer–Emmett–Teller (BET) area, faster electron transfer rate, and better adsorption performance [42]. Furthermore, the activation of PMS by MnO_2_ with different crystal phases (i.e., α-, β-, γ-, and δ-MnO_2_) was reported by Huang et al. [43]. The results demonstrated the important role of crystalline structure and Mn(III) content on the catalytic reactivity of MnO_2_. Saputra et al. investigated the effect of Mn oxidation states (such as MnO, Mn_2_O_3_, Mn_3_O_4_, and MnO_2_) on the activation of PMS for phenol degradation. The results showed that Mn_2_O_3_ has the highest ability on PMS activation among these four manganese oxides [44]. Therefore, the structure of manganese oxides and the content of Mn(III) on the surface of manganese oxides play a critical role in the oxidative and catalytic reactivity of manganese oxides. The performance of MnO_2_ on PDS/PMS activation was well summarized in previous reviews [45,46,47]. However, no attempt has been made to provide a comprehensive review on Mn(III) (oxyhydr)oxides activated PMS/PDS for recalcitrant pollutants removal. 

In light of the above information, this review aims to provide a comprehensive summary of reported Mn(III)-based catalysts in activating PMS/PDS. The structures of commonly used Mn(III) (oxyhydr)oxides (α-Mn_2_O_3_, Mn_3_O_4_, and γ-MnOOH) are first presented, then the effect of structure on the reactivity of Mn(III) (oxyhydr)oxides are discussed. Moreover, the radical and non-radical mechanisms of PMS/PDS activation by a single or combined Mn(III) species are summarized and the influence factors affecting the reactivity of Mn(III) (oxyhydr)oxides are introduced.

We are convinced that this review article will be of significant interest for researchers working on chemical oxidation for water decontamination processes. Finally, we also highlight how the literature lacks information and data that are crucial prior to high-scale applications. 

## 2. Effect of Structure on the Reactivity of Mn(III) (Oxyhydr)Oxides

The oxidative and catalytic performance of manganese oxides can be affected by various structural factors including crystal phases, morphologies, crystal facets, and structural dimensionalities [48]. For instance, Huang et al. reported that δ-MnO_2_ showed higher oxidative activity than α-, β-,γ-, λ- MnO_2_ on bisphenol A oxidation due to the occurrence of more accessible active sites in layered δ-MnO_2_ than other tunnel structured MnO_2_ [49]. The authors also demonstrated the effects of structured MnO_2_ on peroxymonosulfate (PMS) activation, and the low reactivity of δ-MnO_2_ was attributed to its less crystallinity [43]. 

**Figure 1:**
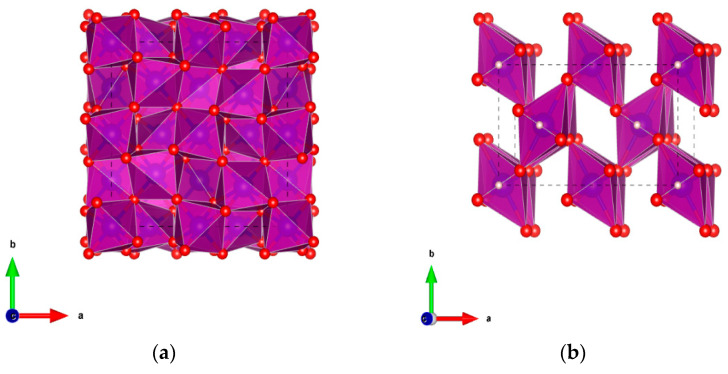

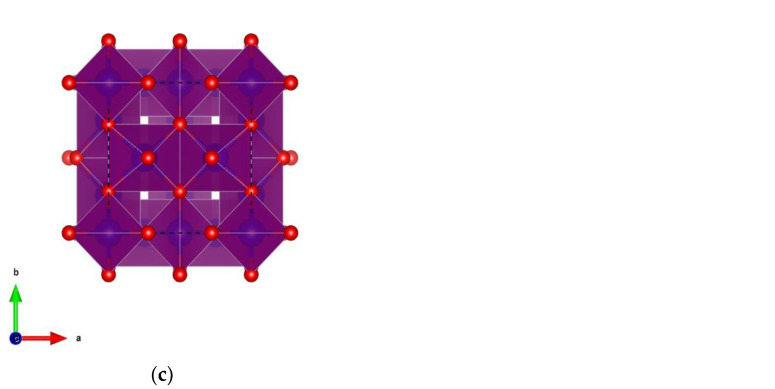
Structural representations of α-Mn_2_O_3_ (**a**), γ-MnOOH (**b**), and (**c**) Mn_3_O_4_. The red, blue, and white balls represent oxygen, manganese, and hydrogen atoms, respectively. The black dashed lines represent the single unit cell. The crystalline parameters of Mn(III) (oxyhydr)oxides were taken from the crystallography open database (COD), and the COD ID of α- Mn_2_O_3_, γ-MnOOH, and Mn_3_O_4_ are 2105791, 1011012, and 1514121, separately [52,53,54].

Crystalline manganese oxides are generally built on the same basic unit [MnO_6_] octahedral with the edges or corners sharing [41]. The commonly reported Mn(III) (oxyhydr)oxides include manganese(III) oxide (α-Mn_2_O_3_), groutite (α-MnOOH), feitknechtite (ꞵ-MnOOH), manganite (γ-MnOOH), and hausmannite (Mn_3_O_4_). The structures covered in the name of Mn(III) (oxyhydr)oxides are summarized in Table 1. Among them, α-Mn_2_O_3_, γ-MnOOH, and Mn_3_O_4_ have attracted increasing attention from the scientific community because of their promising technological applications, such as in catalysis, water treatment, and ion exchange. The crystalline structure of α-Mn_2_O_3_ was recognized as the body-centered cubic bixbyite phase, as shown in Figure 1a. γ-MnOOH possesses a typical (1 × 1) tunnel structure constructed by [MnO_6_] octahedral sharing the corners (Figure 1b). The structure of γ-MnOOH is analogous to that of pyrolusite, except that one-half of the oxygen atoms are replaced by hydroxyl anions compared with pyrolusite. For the crystalline Mn_3_O_4_, it exhibits a normal spinel structure with the formula Mn^2+^(Mn^3+^)_2_O_4_ where the Mn^2+^ and Mn^3+^ ions occupy the tetrahedral and octahedral sites, respectively (Figure 1c).

The influence of structures in the reactivity of common Mn(III) (oxyhydr)oxides is summarized in Table 2. For instance, Saputra et al. investigated the effect of morphology on the oxidation of phenol by Mn_2_O_3_ activated PMS. The results showed that cubic-Mn_2_O_3_ has the highest reactivity on PMS activation in comparison with octahedral- and truncated octahedral-Mn_2_O_3_, and it was due to the high surface area and distinct surface atoms arrangement of cubic-Mn_2_O_3_ [55]. Similarly, Cheng et al. successfully prepared three α-Mn_2_O_3_ in cubic-, truncated octahedral-, and octahedral-structure, and investigated the effect of crystal facets on the combustion of soot [56]. The results show that the soot combustion efficiency followed the order of α-Mn_2_O_3_-cubic > α-Mn_2_O_3_-truncated octahedral > α-Mn_2_O_3_-octahedral. The enhanced reactivity of α-Mn_2_O_3_-cubic was explained by the fact that the exposed (001) surface facets of α-Mn_2_O_3_-cubic have higher amounts of low-coordinated surface oxygen sites, which are capable of facilitating the oxygen activation and improving the surface redox properties. 

In addition to α-Mn_2_O_3_, it was also reported that the oxidative and catalytic performances of Mn_3_O_4_ and γ-MnOOH were affected by their structures. For example, Ji et al. reported that the hexagonal nanoplate Mn_3_O_4_ exhibited superior catalytic performance on diesel soot combustion compared to the octahedral and nanoparticle Mn_3_O_4_, and the finding was explained by the improved amount of surface Mn^4+^ species and surface reactive oxygen species due to the increased fraction of exposed (112) facets in hexagonal nanoplate Mn_3_O_4_ [57]. The effect of morphology was also discovered by Liu et al., which demonstrated that the nanoflake Mn_3_O_4_ (exposure of (001) facet) has the highest oxygen reduction reactivity in comparison to nanoparticle Mn_3_O_4_ and nanorod Mn_3_O_4_ (exposure of (101) facet) [58]. In addition, He et al. investigated the activation of PMS by γ-MnOOH with different shapes, and the results showed that the catalytic activity of γ-MnOOH followed the order of nanowires > multi-branches > nanorods [59]. Different physicochemical parameters, such as specific surface area, Lewis sites, zeta-potential, and redox potential were measured to study the reason for the different catalytic performances of γ-MnOOH with distinct morphologies. It was found that the charge density on the surface played a crucial role in the interfacial reactivity between PMS and γ-MnOOH. In summary, the reactivity of Mn(III) (oxyhydr)oxides on radical precursor activation and pollutant oxidation can be deeply affected by their structures. The desirable morphologies and facets (such as cubic structure with (001) facet exposure) can apparently improve the reactivity of Mn(III) (oxyhydr)oxides. 

**Table 2:** The effect of structures on the reactivity of Mn(III) (oxyhydr)oxides.

Catalysts	Structure	Initial Conditions	Reactivity	Mechanism	Ref. ^1^
α-Mn_2_O_3_	Cubic; octahedral; truncated octahedral	[Catalyst] = 0.4 g/L; [PMS] = 2 g/L; [Phenol] = 25 ppm;	100% of phenol removal by cubic-Mn_2_O_3_ in 60 min	High surface area and surface atoms arrangement of cubic-Mn_2_O_3_	[55]
α-Mn_2_O_3_	Cubic; octahedral; truncated octahedral	[Catalyst] = 4 g/L; [Glycerol] = 20 g/L;	High catalytic activity (0.87 mmol/(h m^2^)) and high selectivity for glycerol (52.6%) was achieved by α-Mn_2_O_3_-truncated octahedral	Co-exposed (001) and (111) facets of α-Mn_2_O_3_-truncated octahedral	[60]
α-Mn_2_O_3_	Octahedral; truncated octahedral	180 mg of catalysts; 500 ppm of NO; 500 ppm of NH_3_; 5% *v/v* of O_2_; N_2_ as balance gas; 36,000 h^−1^ of GSHV;	High NO turnover frequency ((3.6 ± 0.1) × 10^−3^ s^−1^) was achieved by α-Mn_2_O_3_-truncated octahedral at 513 K	The exposure of a small fraction of (001) facets in α-Mn_2_O_3_-truncated octahedral	[61]
α-Mn_2_O_3_	Cubic; octahedral; truncated octahedral	100 mg of catalysts; 10 mg of soot; 5% *v/v* of O_2_; 0.25% *v/v* of NO; N_2_ as balance gas; 9990 h^−1^ of GSHV;	96.3, 89.7, and 85.2% of soot combustion efficiencies were observed with the catalysis of α-Mn_2_O_3_-cubic, -truncated octahedral, -octahedral	The exposed (001) facet of cubic Mn_2_O_3_	[56]
γ-MnOOH	Nanowires; multi-branches; nanorods	[Catalyst] = 0.3 g/L; [PMS] = 12 mM; [2,4-DCP] ^2^ = 100 mg/L; pH = 7;	98%, 88%, and 55% removal of 2,4-DCP was achieved in γ-MnOOH nanowires, multi-branches, and nanorods activated PMS systems, separately	Higher zeta-potential value of nanowires γ-MnOOH	[59]
Mn_3_O_4_	Nano-cubic; nano-plate; nano-octahedral	[Catalyst] = 0.2 g/L; [PMS] = 0.65 mM; [CIP] ^3^ = 10 mg/L; pH = 7.7;	100% CIP removal in 80 min by Mn_3_O_4_ nano-octahedral	Lager surface Mn(IV) contents of Mn_3_O_4_ nano-octahedral	[62]

^1^ Ref.: Reference; ^2^ 2,4-DCP: 2,4-dichlorophenol; ^3^ CIP: ciprofloxacin.

## 3. Mechanisms of PMS/PDS Activation by Mn(III) (Oxyhydr)Oxides

### 3.1. Activation of PMS by Mn(III) (Oxyhydr)Oxides

**Figure 2:**
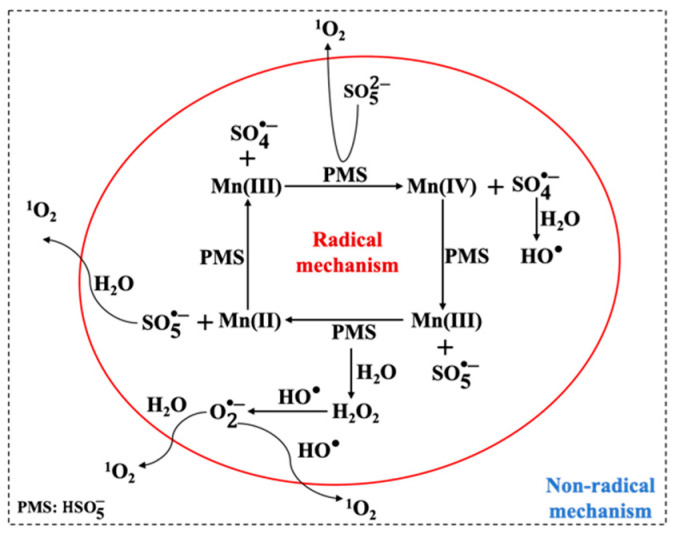
The activation mechanisms of peroxymonosulfate by Mn(III) (oxyhydr)oxides.

The Mn(III) (oxyhydr)oxides/PMS system has been applied for the removal of a number of contaminants, such as phenol, bisphenol A, 2,4-dichlorophenol, ciprofloxacin, and organic dyes [62,63,64,65,66,67]. Different studies involving PMS activation by Mn(III) (oxyhydr)oxides are gathered in Table 3. According to the literature, the efficient degradation of organic pollutants is generally attributed to the generation of active species, such as ${\mathrm{SO}}_{4}^{•-}$, ${\mathrm{HO}}^{•}$, ^1^O_2_. The activation mechanisms of PMS by Mn(III) (oxyhydr)oxides are proposed, as shown in Figure 2. The simultaneous formation of Mn(II) and Mn(IV) and the conversion of Mn ions with different oxidation states explained well the good performance of Mn(III) (oxyhydr)oxides on PMS activation (Equations (1)–(4)) [44]. Except for the above-mentioned processes, the direct generation of ${\mathrm{HO}}^{•}\;$ by Mn(III) activation of PMS was also reported by some researchers (Equation (5)) [62,64,66,68,69,70]. In comparison with ${\mathrm{SO}}_{4}^{•-}$ radical, the ${\mathrm{SO}}_{5}^{•-}$ radical has been regarded as a low oxidative activity for organic pollutants removal due to its low reduction potential (E_0_ = 1.10 V vs. NHE) [71]. Nevertheless, the transformation from ${\mathrm{SO}}_{5}^{•-}$ to ${\mathrm{SO}}_{4}^{•-}$ in Mn(III) (oxyhydr)oxides/PMS system still makes some contribution to the degradation of organic pollutants (Equation (6)) [72]. In addition, the conversion from ${\mathrm{SO}}_{4}^{•-}$ to ${\mathrm{HO}}^{•}$ in water should not be neglected (Equation (7)), especially, when the solution is in the alkaline environment (Equation (8)) [73].

(1) $$\mathrm{Mn}(\mathrm{III})+{\mathrm{HSO}}_{5}^{-}\to \;\mathrm{Mn}(\mathrm{IV})+{\mathrm{SO}}_{4}^{•-}+{\mathrm{OH}}^{-}$$

(2) $$\mathrm{Mn}(\mathrm{III})+{\mathrm{HSO}}_{5}^{-}\to \;\mathrm{Mn}(\mathrm{II})+{\mathrm{SO}}_{5}^{•-}+H^{+}$$

(3) $$\mathrm{Mn}(\mathrm{II})+{\mathrm{HSO}}_{5}^{-}\to \;\mathrm{Mn}(\mathrm{III})+{\mathrm{SO}}_{4}^{•-}+{\mathrm{OH}}^{-}$$

(4) $$\mathrm{Mn}(\mathrm{IV})+{\mathrm{HSO}}_{5}^{-}\to \;\mathrm{Mn}(\mathrm{III})+{\mathrm{SO}}_{5}^{•-}+H^{+}$$

(5) $$\mathrm{Mn}(\mathrm{III})+{\mathrm{HSO}}_{5}^{-}\to \;\mathrm{Mn}(\mathrm{IV})+{\mathrm{SO}}_{4}^{2-}+{\mathrm{HO}}^{•}$$

(6) $${\mathrm{SO}}_{5}^{•-}+{\mathrm{SO}}_{5}^{•-}\to \;O_{2}+2\;{\mathrm{SO}}_{4}^{•-}$$

(7) $${\mathrm{SO}}_{4}^{•-}\;+H_{2}O\to {\mathrm{SO}}_{4}^{2-}+{\mathrm{HO}}^{•}+H^{+}$$

(8) $${\mathrm{SO}}_{4}^{•-}+{\mathrm{OH}}^{-}\to {\mathrm{SO}}_{4}^{2-}+{\mathrm{HO}}^{•}$$

In addition to the active radicals, the generation of non-radical species (such as ^1^O_2_) in the Mn(III) (oxyhydr)oxides-activated PMS system was also reported. For example, He et al. demonstrated the contribution of ^1^$O_{2}$ for the degradation of 2,4-dichlorophenol in the γ-MnOOH/PMS system. The generation of ^1^$O_{2}$ was attributed to two pathways including the decomposition of PMS and the reaction of $O_{2}^{•-}$ with ${\mathrm{HO}}^{•}$ (Equations (9) and (10)) [59,74,75]. Chen et al. synthesized one new Mn_3_O_4_ nanodots-g-C_3_N_4_ nanosheet (Mn_3_O_4_/CNNS) and investigated its performance on PMS activation for 4-chlorophenol (4-CP) degradation [76]. The chemical scavenging tests and electron spin resonance (ESR) experiments confirmed the contribution of ^1^$O_{2}$ for the removal of 4-CP. Furthermore, new pathways for the formation of ^1^$O_{2}$ were reported in the Mn_3_O_4_/CNNS/PMS system. As shown in Equations (11)–(16), the reaction between ${\mathrm{SO}}_{5}^{•-}$ and $H_{2}O$ and the combination of $O_{2}^{•-}\;$ with $H_{2}O$ can contribute to the formation of ^1^$O_{2}$ [76].

(9) $${\mathrm{HSO}}_{5}^{-}+{\mathrm{SO}}_{5}^{2-}\to {\;\mathrm{HSO}}_{4}^{-}+{\mathrm{SO}}_{4}^{2-}+{\;}^{1}O_{2}$$

(10) $$O_{2}^{•-}+{\mathrm{HO}}^{•}\to \;{\;}^{1}O_{2}+{\mathrm{OH}}^{-}$$

(11) $$2\;{\mathrm{SO}}_{5}^{•-}+H_{2}O\to {\;2\;\mathrm{HSO}}_{4}^{-}+1.5{\;}^{1}O_{2}$$

(12) $${\mathrm{HSO}}_{5}^{-}+H_{2}O\to {\;\mathrm{HSO}}_{4}^{-}+H_{2}O_{2}$$

(13) $$H_{2}O_{2}\to H^{+}+{\mathrm{HO}}_{2}^{-}\;\;\;\mathrm{pKa}=11.6$$

(14) $$H_{2}O_{2}+{\mathrm{HO}}^{•}\to H_{2}O+{\mathrm{HO}}_{2}^{•}$$

(15) $${\mathrm{HO}}_{2}^{•}\to H^{+}+O_{2}^{•-}\;\;\;\mathrm{pKa}=4.88$$

(16) $$2\;O_{2}^{•-}+2\;H_{2}O\to {\;H}_{2}O_{2}+{\;}^{1}O_{2}+2\;{\mathrm{OH}}^{-}$$

Currently, the Mn-based oxide composites have attracted increasing attention due to their various advantages, such as more oxygen vacancies, higher surface oxygen mobility, and enforced synergistic effects. For instance, Chen et al. prepared the Fe_2_O_3_/Mn_2_O_3_ composite and studied its activity on PMS activation for tartrazine (TTZ) degradation. The results showed that 97.3% removal of TTZ was achieved in 30 min in the Fe_2_O_3_/Mn_2_O_3_/PMS system. The efficient degradation of TTZ originated from the generation of active species (e.g., ${\mathrm{SO}}_{4}^{•-}$, ${\mathrm{HO}}^{•}$) and the synergistic effect between iron and manganese ions [77]. The γ-MnOOH-coated nylon membrane was synthesized and applied in the activation of PMS towards the removal of 2,4-dichlorophenol (2,4-DCP). The deep removal of 2,4-DCP was explained by the synergetic “trap-and-zap” process, which improved the stability and catalytic reactivity of γ-MnOOH [63]. In conclusion, the activation of PMS by Mn(III) (oxyhydr)oxides, including pure Mn(III) oxides and Mn(III) containing composites, is favorable. The degradation of various pollutants in the Mn(III) (oxyhydr)oxides/PMS system can be achieved through the generation of active radicals and non-radical species.

**Table 3:** Summary of PMS activation by Mn(III) (oxyhydr)oxides.

Catalysts	Pollutant	Initial Conditions	Reactivity	Active Species	Ref.
Mn_2_O_3_	Phenol	[Catalyst] = 0.4 g/L; [PMS] = 2 g/L; [Phenol] = 25 mg/L;	100% removal of phenol in 60 min	${\mathrm{SO}}_{4}^{•-}$	[44]
Mn_3_O_4_	Phenol	[Catalyst] = 0.4 g/L; [PMS] = 2 g/L; [Phenol] = 25 mg/L;	100% removal of phenol in 20 min	${\mathrm{SO}}_{4}^{•-}$	[78]
Mn_3_O_4_ nanoparticle	Methylene blue (MB)	[Catalyst] = 0.12 g/L; [PMS] = 0.94 g/L; [MB] = 62 mg/L; pH = 4;	86.71% removal of MB in 20 min	${\mathrm{SO}}_{4}^{•-}$	[64]
Mn_3_O_4_ nano-octahedral	Ciprofloxacin (CIP)	[Catalyst] = 0.2 g/L; [PMS] = 0.65 mM; [CIP] = 10 mg/L; pH = 7.7;	100% removal of CIP in 80 min	${\mathrm{SO}}_{4}^{•-}$ ${\mathrm{HO}}^{•}$	[62]
yolk-shell Mn_3_O_4_	Bisphenol A (BPA)	[Catalyst] = 0.1 g/L; [PMS] = 0.3 g/L; [BPA] = 10 mg/L; pH = 5.3;	87.7% of removal of BPA in 60 min	${\mathrm{SO}}_{4}^{•-}$ ${\mathrm{HO}}^{•}$	[67]
3D hierarchical Mn_3_O_4_	Phenol	[Catalyst] = 0.2 g/L; [PMS] = 6.5 mM; [Phenol] = 20 ppm; pH = 6.8;	100% removal of phenol in 60 min	${\mathrm{SO}}_{4}^{•-}$ ${\mathrm{HO}}^{•}$	[66]
dumbbell-like Mn_2_O_3_	Rhodamine B (RhB)	[Catalyst] = 0.25 g/L; [PMS] = 0.75 g/L; [RhB] = 10 mg/L;	100% of removal of RhB in 30 min	${\mathrm{SO}}_{4}^{•-}$ ${\mathrm{HO}}^{•}$ $O_{2}^{•-}$ ^1^ $O_{2}$	[65]
α-Mn_2_O_3_-cubic	Phenol	[Catalyst] = 0.4 g/L; [PMS] = 2 g/L; [Phenol] = 25 ppm;	100% removal of phenol in 1 h	${\mathrm{SO}}_{4}^{•-}$	[55]
γ-MnOOH nanowire	2,4-dichlorophenol (2,4-DCP)	[Catalyst] = 0.3 g/L; [PMS] = 12 mM; [2,4-DCP] = 100 mg/L; pH = 7;	98% removal of 2,4-DCP in 6 h	${\mathrm{SO}}_{4}^{•-}$ ${\mathrm{HO}}^{•}$ $O_{2}^{•-}$ ^1^ $O_{2}$	[59]
MnOOH@nylon	2,4-DCP	[Catalyst] = 0.76; mg/cm2; [PMS] = 138 mg/L; [2,4-DCP] = 25 mg/L; pH = 6.0-6.4;	97.9% removal of 2,4-DCP in 2 h	${\mathrm{SO}}_{4}^{•-}$ ${\mathrm{HO}}^{•}$ $O_{2}^{•-}$ ^1^ $O_{2}$	[63]
γ-MnOOH-rGO	Bentazone	[Catalyst] = 0.075 g/L; [PMS] = 0.615 g/L; [Bentazone] = 10 mg/L; pH = 7; sunlight;	96.1% removal of Bentazone in 90 min	${\mathrm{HO}}^{•}$ ^1^ $O_{2}$	[79]
Ce-Mn_2_O_3_	2,4-DCP	[Catalyst] = 0.2 g/L; [PMS] = 1.0 g/L; [2,4-DCP] = 50 mg/L; pH = 7;	100% removal of 2,4-DCP in 90 min	${\mathrm{SO}}_{4}^{•-}$ ${\mathrm{HO}}^{•}$ ^1^ $O_{2}$	[80]
Mn_3_O_4_-GO	Orange II	[Catalyst] = 50 mg/L; [PMS] = 1.5 g/L; [Orange II] = 30 mg/L; pH = 7.0;	100% removal of Orange II in 120 min	${\mathrm{SO}}_{4}^{•-}$	[81]
Fe_2_O_3_/Mn_2_O_3_	Tartrazine (TTZ)	[Catalyst] = 0.6 g/L; [PMS] = 0.8 g/L; [TTZ] = 10 mg/L; pH = 6.89;	97.3% removal of TTZ in 30 min	${\mathrm{SO}}_{4}^{•-}$ ${\mathrm{HO}}^{•}$	[77]
Mn_2_O_3_@Mn_5_O_8_	4-chlorophenol (4-CP)	[Catalyst] = 0.3 g/L; [PMS] = 1.5 mM; [4-CP] = 80 ppm;	100% removal of 4-CP in 60 min	${\mathrm{SO}}_{4}^{•-}$ ${\mathrm{HO}}^{•}$ $O_{2}^{•-}$ ^1^ $O_{2}$	[82]
Mn_3_O_4_-MnO_2_	CIP	[Catalyst] = 0.1 g/L; [PMS] = 1 mM; [CIP] = 50 μM; pH = 7.0 ± 0.1;	97.6% removal of CIP in 25 min	${\mathrm{SO}}_{4}^{•-}$ ${\mathrm{HO}}^{•}$	[68]
Mn_3_O_4_/MOF	RhB	[Catalyst] = 0.4 g/L; [PMS] = 0.3 g/L; [RhB] = 10 mg/L; pH = 5.18;	98% removal of RhB in 60 min	${\mathrm{SO}}_{4}^{•-}$ ${\mathrm{HO}}^{•}$	[69]
Fe_3_O_4_/Mn_3_O_4_/GO	MB	[Catalyst] = 100 mg/L; [PMS] = 0.3 g/L; [MB] = 50 mg/L; pH = 7;	98.8% removal of MB in 30 min	${\mathrm{SO}}_{4}^{•-}$ ${\mathrm{HO}}^{•}$	[83]
Mn_3_O_4_/CNNS-150	4-CP	[Catalyst] = 0.3 g/L; [PMS] = 1 mM; [4-CP] = 50 mg/L; pH = 6.89;	100% removal of 4-CP in 60 min	^1^ $O_{2}$	[76]
α-Mn_2_O_3_@α-MnO_2_-350	Phenol	[Catalyst] = 0.4 g/L; [PMS] = 2.0 g/L; [Phenol] = 25 mg/L; pH = 3-3.5;	100% removal of phenol in 25 min	${\mathrm{SO}}_{4}^{•-}$ ${\mathrm{HO}}^{•}$	[84]
α-Mn_2_O_3_@α-MnO_2_-500	Phenol	[Catalyst] = 0.15 g/L; [PMS] = 1 mM; [Phenol] = 25 ppm;	100% removal of phenol in 70 min	${\mathrm{SO}}_{4}^{•-}$ ${\mathrm{HO}}^{•}$ ^1^ $O_{2}$	[85]
CuS/Fe_2_O_3_/Mn_2_O_3_	CIP	[Catalyst] = 0.6 g/L; [PMS] = 0.6 g/L; [CIP] = 10 mg/L; pH = 5.84;	88% removal of CIP in 120 min	${\mathrm{SO}}_{4}^{•-}$ ${\mathrm{HO}}^{•}$	[86]

### 3.2. Activation of PDS by Mn(III) (Oxyhydr)Oxides

**Figure 3:**
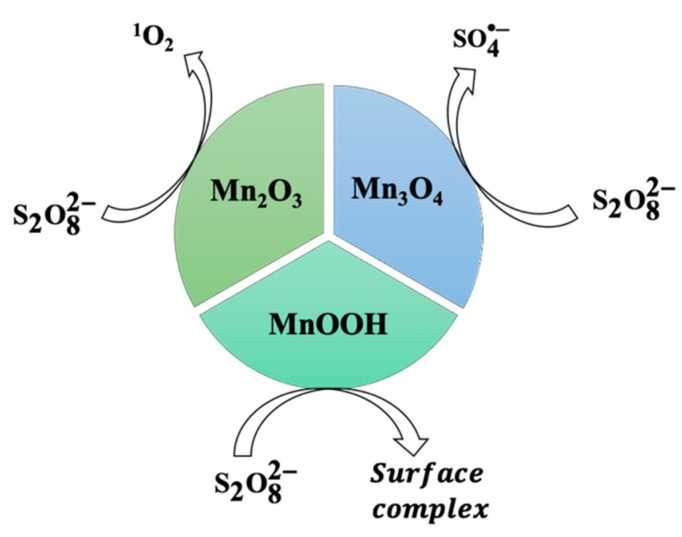
The activation mechanisms of peroxydisulfate by various Mn(III) (oxyhydr)oxides.

Single or combined Mn(III) (oxyhydr)oxides have been employed to activate PDS to remove different organic pollutants, such as phenol, p-chloroaniline (PCA), 2,4-dichlorophenol (2,4-DCP), and organic dyes (Table 4). The activation pathway of PDS varies with the different types of Mn(III) (oxyhydr)oxides (Figure 3). For example, Shabanloo et al. reported the generation of active ${\mathrm{SO}}_{4}^{•-}\;$ radicals in the nano-Mn_3_O_4_/PDS system [87]. Since both Mn(II) and Mn(III) species are identified in the Mn_3_O_4_ structure, the formation of ${\mathrm{SO}}_{4}^{•-}$ was mainly attributed to the activation of PDS by Mn(II) (Equation (17)). In contrast, the persulfate radical ($S_{2}O_{8}^{•-}$) was produced by the reaction of PDS and Mn(III) (Equation (18)). For the system of Mn_2_O_3_/PDS, it is believed that the singlet oxygen (^1^$O_{2}$) was the primary active species that was responsible for the degradation of organic pollutants [88]. As demonstrated by Khan et al., one complex ≡ Mn(III/IV)-${\mathrm{OS}}_{2}O_{7}^{-}$ was formed between PDS and Mn_2_O_3_ through the inner-sphere interaction. Then, another $S_{2}O_{8}^{2-}$ was decomposed by ≡ Mn(III/IV)-${\mathrm{OS}}_{2}O_{7}^{-}$ to generate ${\mathrm{HO}}_{2}^{•}/O_{2}^{•-}\;$ radicals. The ^1^$O_{2}$ was finally formed from the direct oxidation of $O_{2}^{•-}\;$ by ≡ Mn(IV)-${\mathrm{OS}}_{2}O_{7}^{-}$ or the recombination of ${\mathrm{HO}}_{2}^{•}$ and $O_{2}^{•-}$ (Equations (19)–(20)). The pathway of ^1^$O_{2}$ formation in the system of A-Mn_2_O_3_/PDS is comparable to the approach of producing ^1^$O_{2}$ in the β-MnO_2_/PDS system in which the important metastable manganese intermediate was first formed through the complex reaction between the hydroxyl group (-OH) and cleavaged $S_{2}O_{8}^{2-}$ [41]. Therefore, the hydroxyl group on the surface of manganese oxides plays a significant role in PDS activation.

(17) $$\equiv \mathrm{Mn}(\mathrm{II})+S_{2}O_{8}^{2-}\to \equiv \mathrm{Mn}(\mathrm{III})+{\mathrm{SO}}_{4}^{•-}+{\mathrm{SO}}_{4}^{2-}.$$

(18) $$\equiv \mathrm{Mn}(\mathrm{III})+S_{2}O_{8}^{2-}\to \equiv \mathrm{Mn}(\mathrm{II})+S_{2}O_{8}^{•-}$$

(19) $$\equiv \mathrm{Mn}(\mathrm{IV})-{\mathrm{OS}}_{2}O_{7}^{-}+O_{2}^{•-}+{\mathrm{OH}}^{-}\to \equiv \mathrm{Mn}(\mathrm{III})-{\mathrm{OH}}^{-}+2\;{\mathrm{SO}}_{4}^{2-}+{\;}^{1}O_{2}$$

(20) $$O_{2}^{•-}+{\mathrm{HO}}_{2}^{•}\to \;{\;}^{1}O_{2}+HO_{2}^{-}$$

**Figure 4:**
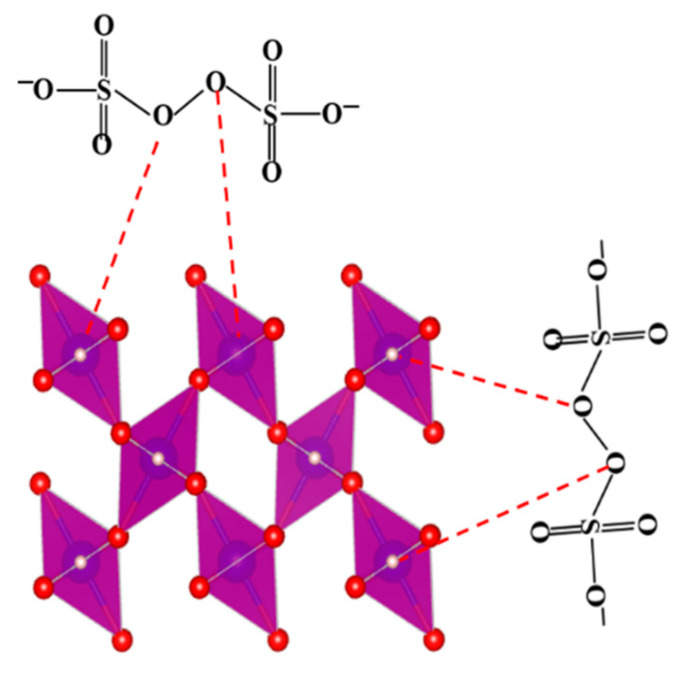
The diagram of PDS activation on the surface of γ-MnOOH. The red, blue, and white balls in the structure of γ-MnOOH represent the oxygen, manganese, and hydrogen atoms, respectively. The COD ID of γ-MnOOH is 1011012 [54].

In comparison with Mn_3_O_4_ and Mn_2_O_3_, γ-MnOOH presents more -OH groups on the surface, leading to the high efficiency in PDS activation. For instance, Li et al. reported that γ-MnOOH exhibited higher reactivity in PDS activation for phenol oxidation in comparison with Mn_2_O_3_ and Mn_3_O_4_ [89]. The authors reported that the degradation efficiency of phenol in the γ-MnOOH/PDS system was pH-dependent. Under the basic condition (pH 11), phenol was efficiently removed due to the generation of ${\mathrm{SO}}_{4}^{•-}$ and ${\mathrm{HO}}^{•}$ radicals. However, at pH 3 and 7, the oxidative intermediate (≡Mn(III)−O3−SOOSO3−) was believed to be responsible for the removal of phenol. Although the mentioned report explained well the oxidation performance of γ-MnOOH/PMS for phenol removal, the information regarding the mechanism of PDS activation on the surface of γ-MnOOH was not given in detail. Considering this, Xu et al. conducted a further investigation focusing on the catalytic mechanism of PDS by γ-MnOOH [90]. Based on the results of chemical scavenging and ESR experiments, a non-radical mechanism was proposed. Generally, the non-radical mechanism in PS activation was attributed to three aspects—the generation of ^1^$O_{2}$, the electron transfer process, and the catalyst surface-activated intermediates [91,92,93,94,95]. However, the ^1^$O_{2}\;$ production and electron transfer process mechanism were excluded according to the results of ESR and linear sweep voltammetry (LSV) experiments. Therefore, the γ-MnOOH surface-activated PDS molecules were verified as the main active species for the degradation of PCA. Figure 4 shows the formation of active PDS molecules on the surface of γ-MnOOH.

The activation of PDS by Mn(III) (oxyhydr)oxide composites for pollutant degradation was also reported [96,97,98]. For instance, Liu et al. synthesized the carbon-coated Mn_3_O_4_ composite (Mn_3_O_4_/C) and investigated the reactivity in the presence of PDS for 2,4-dichlorophenol (2,4-DCP) degradation [96]. The results showed that 95% of 2,4-DCP removal was reached in 140 min and the enhanced degradation was attributed to the existence of the defective edges of the carbon layer, which facilitated the attraction and activation of PDS. Rizal et al. prepared Ag/Mn_3_O_4_ and Ag/Mn_3_O_4_/graphene composites and studied the degradation efficiency of methylene blue (MB) by the synthesized catalysts activated PDS in the presence of visible light [97]. The results showed that 40 mg/L of MB was completely removed in 30 min by the system of Ag/Mn_3_O_4_/graphene + PDS under visible light. The enhanced degradation of MB was attributed to the hampered electron-hole recombination due to the loading of Ag and graphene. Furthermore, the studies regarding the application of modified Mn_2_O_3_ in oxidants (such as PMS, $H_{2}O_{2}$) activation for contaminants removal were also reported [84,99,100,101]. For example, Saputra et al. prepared an egg-shaped core/shell α-Mn_2_O_3_@α-MnO_2_ catalyst via a hydrothermal process and investigated the catalytic activity of α-Mn_2_O_3_@α-MnO_2_ in heterogeneous Oxone® activation for phenol degradation [84]. The loaded α-MnO_2_ improved the generation of Mn(III) species through the reaction with PMS. The amount of ${\mathrm{SO}}_{4}^{•-}$ and ${\mathrm{HO}}^{•}$ was then increased leading to the enhanced degradation of phenol. The efficient degradation of organic dye pollutants (such as Rhodamine B (RhB) and Congo Red (CR)) by bimetallic Mn_2_O_3_-Co_3_O_4_/carbon catalyst activated Fenton-like reaction was also reported [100]. The superior reactivity of Mn_2_O_3_-Co_3_O_4_/C catalyst in $H_{2}O_{2}$ activation for RB and CR degradation was attributed to the good synergistic effect between Co_3_O_4_ and Mn_2_O_3_ as well as the interaction between metal oxides and carbon. However, the investigation regarding the activation of PDS by modified α-Mn_2_O_3_ has been less reported. The same effect was also observed for the γ-MnOOH-based composites. This might be attributed to the distinct activation way of PDS by α-Mn_2_O_3_ or γ-MnOOH compared with Mn_3_O_4_.

In summary, Mn_3_O_4_ can activate PDS to generate ${\mathrm{SO}}_{4}^{•-}$ through radical mechanisms, while the activation of PDS by α-Mn_2_O_3_ and γ-MnOOH is processed in a non-radical mechanism with the generation of ^1^O_2_ and catalyst surface-activated PDS substances. For the activation of PDS by Mn(III) (oxyhydr)oxides composites, the Mn_3_O_4_-based composites have shown good catalytic performance in PDS activation for pollutant degradation. In comparison, the activation of PDS by modified α-Mn_2_O_3_ or γ-MnOOH catalysts needs to be further investigated.

**Table 4:** Summary of PDS activation by Mn(III) (oxyhydr)oxides.

Catalysts	Pollutant	Initial Conditions	Reactivity	Active Species	Ref.
γ-MnOOH	P-chloroaniline (PCA)	[Catalyst] = 0.4 g/L; [PDS] = 2.5 mM; [PCA] = 0.5 mM; pH = 4.2;	100% removal of PCA in 180 min	γ-MnOOH-PDS complex	[90]
A-Mn_2_O_3_	Phenol	[Catalyst] = 0.2 g/L; [PDS] = 2 mM; [Phenol] = 12 ppm; pH = 3.2;	100% removal of phenol in 70 min	^1^ $O_{2}$	[88]
Mn_3_O_4_ nanoparticle	Acid Blue 113 (AB113)	[Catalyst] = 57.69 mg/L; [PDS] = 61.46 mg/L; [AB113] = 50 mg/L; pH = 3;	96.7% removal of AB113 in 60 min	${\mathrm{SO}}_{4}^{•-}$ ${\mathrm{HO}}^{•}$	[102]
γ-MnOOH	Phenol	[Catalyst] = 1 g/L; [PDS] = 2 g/L; [Phenol] = 100 mg/L; pH = 7;	91.86% removal of phenol in 360 min	γ-MnOOH-PDS complex	[89]
Nano-Mn_3_O_4_	Furfural	[Catalyst] = 1.2 g/L; [PDS] = 6.34 mM; [Furfural] = 50 mg/L; pH = 4.82;	91.14% of furfural removal in 60 min	${\mathrm{SO}}_{4}^{•-}$	[87]
Ag/Mn_3_O_4_-5 G	MB	[Catalyst] = 0.5 g/L; [PDS] = 12 mM; [MB] = 40 mg/L; pH = 3; visible-light;	100% of MB removal in 30 min	${\mathrm{SO}}_{4}^{•-}$ ${\mathrm{HO}}^{•}$	[97]
Mn_2_O_3_/Mn_3_O_4_/MnO_2_-10	Orange II	[Catalyst] = 0.4 g/L; [PDS] = 2 g/L; [Orange II] = 20 mg/L;	95% removal of Orange II in 50 min	${\mathrm{SO}}_{4}^{•-}$ ${\mathrm{HO}}^{•}$	[103]
0.5-Mn_3_O_4_/C-T4	2,4-DCP	[Catalyst] = 0.2 g/L; [PDS] = 2 g/L; [2,4-DCP] = 100 mg/L; pH = 6.37;	95% removal of 2,4-DCP in 140 min	${\mathrm{SO}}_{4}^{•-}$ ${\mathrm{HO}}^{•}$ ^1^ $O_{2}$	[96]
γ-Fe_2_O_3_/Mn_3_O_4_	RhB	[Catalyst] = 50 mg/L; [PDS] = 50 mg/L; [RhB] = 10 mg/L; pH = 4.5;	97.5% removal of RhB in 150 min	${\mathrm{SO}}_{4}^{•-}$ ${\mathrm{HO}}^{•}$	[98]

## 4. Influence Factors for Mn(III) (Oxyhydr)Oxides Reactivity

### 4.1. The Effect of pH

The Mn(III) (oxyhydr)oxides-mediated activation of PDS/PMS can be affected by solution pH in different ways. For example, influencing the property of charge on the surface of the catalysts, changing the ionic forms of PDS/PMS and pollutant molecules, as well as altering the reduction potential of active radicals.

First, the solution pH can affect the interaction between catalyst and PDS/PMS and pollutants through changing the electrostatic effect. The point of zero charges (PZC) of the catalyst and the acid dissociation constant (pKa) of radical precursors and contaminants are two important parameters that are used to recognize the charge type on the surface of the catalysts and the ionic situation of oxidants and pollutants in solution. For instance, when the solution pH is equal to the PZC value of the catalyst, the amounts of positive and negative charges on the surface of the catalyst are equal (i.e., the surface charge of the catalyst is zero). When the solution pH is higher than the PZC value of the catalyst, the surface charges of the catalyst are negative. On the contrary, if the solution pH is lower than the catalyst PZC value, the surface of the catalyst will be positively charged [104]. The same situation is suitable for the analysis of the ionic form of oxidants and pollutants. The PZC values of commonly used Mn(III) (oxyhydr)oxides and the pKa values of PMS/PDS, and some typical pollutants, are summarized in Table 5. The impacts of solution pH on the interaction between Mn(III) (oxyhydr)oxides and PDS/PMS and pollutants have been reported. For example, Zhao et al. reported that the adsorption and degradation of ciprofloxacin (CIP) by the synthesized Mn_3_O_4_-MnO_2_ composite were facilitated at neutral pH solution [68]. The results were explained by the enhanced electrostatic attraction between Mn_3_O_4_-MnO_2_ and CIP. The PZC value of the Mn_3_O_4_-MnO_2_ composite was measured at 2.5; thus, in the solution pH 7, the surface of the catalyst was negatively charged. In comparison, the pKa of CIP was 8.7–10.58, leading to the formation of positively charged CIP ions in the neutral pH solution. Therefore, the electrostatic attraction between the negative catalyst and the positive CIP improved, resulting in a facilitating degradation of CIP. The same phenomenon was also reported in the studies of PDS activation by γ-MnOOH/α-Mn_2_O_3_ for pollutant degradation [88,90].

Second, the transformation of radicals also influenced the reactivity of Mn(III) (oxyhydr)oxides for pollutant degradation. For instance, the reported conversion of ${\mathrm{SO}}_{4}^{•-}$ to ${\mathrm{HO}}^{•}$ under the basic solution (as shown in (Equation (8)) can have a significant impact. Since the reduction potential value of ${\mathrm{HO}}^{•}$ under natural pH is lower than that in acidic solution (1.8 vs. 2.7V) [105], and the lifetime of ${\mathrm{HO}}^{•}$ is shorter than ${\mathrm{SO}}_{4}^{•-}$ (20 ns vs. 30–40 μs) [106]; thus, the transformation from ${\mathrm{SO}}_{4}^{•-}$ (E = 2.6 V) to ${\mathrm{HO}}^{•}$ under alkaline solution might lead to a decrease of pollutant degradation. In addition, the leaching of Mn^2+^ from Mn(III) (oxyhydr)oxides in an acidic condition also should be taken into consideration for the activation of sulfate compounds (PMS/PDS).

**Table 5:** The PZC values of Mn(III) (oxyhydr)oxides and pKa values of PMS/PDS and pollutants.

Catalysts	PZC	Reference
α-Mn_2_O_3_	4.7	[88,107]
γ-MnOOH	3.4	[90]
Mn_3_O_4_	5.6–7.34	[68,87,102]
**Oxidants**	**pKa**	**Reference**
PMS	9.4	[108]
PDS	−3.5	[109]
**Pollutants**	**pKa**	**Reference**
Phenol	9.98	[110]
Bisphenol A	9.6–10.2	[111]
2,4-dichlorophenol	9.4	[82]
Ciprofloxacin	8.70–10.58	[68,112]
p-Chloroaniline	4.2	[90,113]
4-Chlorophenol	9.29	[114]
Orange II	11.4	[103]

### 4.2. The Effect of Inorganic Anions

Inorganic anions are ubiquitous in various aquatic compartments. It is reported that inorganic anions can suppress the degradation of pollutants in Mn(III) (oxyhydr) oxides-activated PMS/PDS systems through competing with pollutants for radicals. Thus, to evaluate the applicability of the Mn(III) (oxyhydr)oxides + PMS/PDS system in different water matrices, the influence of inorganic anions on the removal of pollutants has been investigated by many researchers [63,79,86,88,97]. In this section, the effect of inorganic anions, such as carbonate/bicarbonate ions (${\mathrm{CO}}_{3}^{2-}$/${\mathrm{HCO}}_{3}^{-}$), chloride ions (${\mathrm{Cl}}^{-}$), and nitrate (${\mathrm{NO}}_{3}^{-}$)/nitrite ions (${\mathrm{NO}}_{2}^{-}$) on the reactivity of Mn(III) (oxyhydr)oxides was summarized.

Carbonate (${\mathrm{CO}}_{3}^{2-}$)/bicarbonate (${\mathrm{HCO}}_{3}^{-}$) can react with ${\mathrm{SO}}_{4}^{•-}$ and ${\mathrm{HO}}^{•}$ to generate less reactive carbonate radical (${\mathrm{CO}}_{3}^{•-}$) and bicarbonate radical (${\mathrm{HCO}}_{3}^{•}$) (Equations (21)–(25)) leading to the inhibited degradation of pollutants [115]. However, although the redox potential of ${\mathrm{CO}}_{3}^{•-}$ is low (1.59 V vs. NHE), it can still selectively degrade some organic pollutants with a reaction rate of 10^3^–10^9^ M^−1^s^−1^ [116,117]. In addition, the presence of carbonate and bicarbonate ions can affect the stability of oxidants. For example, PDS can be activated by ${\mathrm{HCO}}_{3}^{-}$ to generated percarbonate (${\mathrm{HCO}}_{4}^{-}$) (Equation (26)) [118]. Similarly, PMS can be catalyzed by both ${\mathrm{CO}}_{3}^{2-}$ and ${\mathrm{HCO}}_{3}^{-}$ to form active radicals and ${\mathrm{HCO}}_{4}^{-}$ (Equations (27)–(29)). Furthermore, the solution pH can be changed in the presence of carbonate/bicarbonate ions, which can affect the reactivity of Mn(III) (oxyhydr)oxides in PMS/PDS activation as discussed in Section 4.1.

(21) $${\mathrm{SO}}_{4}^{•-}+{\mathrm{CO}}_{3}^{2-}\;\to \;{\mathrm{SO}}_{4}^{2-}+{\mathrm{CO}}_{3}^{•-}$$

(22) $${\mathrm{SO}}_{4}^{•-}+{\mathrm{HCO}}_{3}^{-}\;\to \;{\mathrm{SO}}_{4}^{2-}+{\mathrm{HCO}}_{3}^{•}$$

(23) $${\mathrm{HO}}^{•}+{\mathrm{CO}}_{3}^{2-}\;\to \;{\mathrm{OH}}^{-}+{\mathrm{CO}}_{3}^{•-}$$

(24) $${\mathrm{HO}}^{•}+{\mathrm{HCO}}_{3}^{-}\;\to H_{2}O+{\mathrm{HCO}}_{3}^{•}$$

(25) $${\mathrm{HCO}}_{3}^{•}=H^{+}+{\mathrm{CO}}_{3}^{•-}\;\mathrm{pKa}=9.6$$

(26) $$S_{2}O_{8}^{2-}+{\mathrm{HCO}}_{3}^{-}+2\;{\mathrm{OH}}^{-}\;\to \;{\mathrm{HCO}}_{4}^{-}+2\;{\mathrm{SO}}_{4}^{2-}+H_{2}O$$

(27) $${\mathrm{HSO}}_{5}^{-}+{\mathrm{CO}}_{3}^{2-}+H^{+}\;\to \;{\mathrm{SO}}_{4}^{•-}+2\;{\mathrm{OH}}^{-}+{\mathrm{CO}}_{2}$$

(28) $${\mathrm{HSO}}_{5}^{-}+{\mathrm{HCO}}_{3}^{-}\;\to \;{\mathrm{SO}}_{4}^{•-}+2\;{\mathrm{OH}}^{-}\;+{\mathrm{CO}}_{2}$$

(29) $${\mathrm{HSO}}_{5}^{-}+{\mathrm{HCO}}_{3}^{-}\;\to {\mathrm{SO}}_{4}^{2-}+{\mathrm{HCO}}_{4}^{-}+H^{+}$$

Chloride ion (${\mathrm{Cl}}^{-}$) exists widely in various water bodies including surface water, groundwater, and industrial wastewater [119]. The influence of ${\mathrm{Cl}}^{-}$ on the degradation of organic pollutants by sulfate radical-based AOPs (SR-AOP) was reported by previous studies [120,121,122,123]. Generally, ${\mathrm{Cl}}^{-}$ can react with ${\mathrm{SO}}_{4}^{•-}$ to generate ${\mathrm{Cl}}^{•}$, which can react with another ${\mathrm{Cl}}^{-}$ to form ${\mathrm{Cl}}_{2}^{•-}$ (Equations (30)–(31)) [122]. Both ${\mathrm{Cl}}^{•}$ and ${\mathrm{Cl}}_{2}^{•-}$ have low reduction potentials (E_0_ = 2.4 and 2.0 V) in comparison with ${\mathrm{SO}}_{4}^{•-}$, thus the consumption of ${\mathrm{SO}}_{4}^{•-}$ by ${\mathrm{Cl}}^{-}$ leads to the decrease of organic pollutant degradation [124,125]. However, ${\mathrm{Cl}}^{•}$ was believed to own higher selectivity on electron-rich compounds than nonselective ${\mathrm{SO}}_{4}^{•-}$, which can offset the negative effect of ${\mathrm{Cl}}^{-}$ on ${\mathrm{SO}}_{4}^{•-}$ [126]. Therefore, the conflicting effect of ${\mathrm{Cl}}^{-}\;$ on organic pollutants in SR-AOP might be attributed to the different reactivity of pollutants with ${\mathrm{Cl}}^{•}$ and ${\mathrm{Cl}}_{2}^{•-}$. In addition, the reactivity of ${\mathrm{HO}}^{•}\;$ can also be suppressed by ${\mathrm{Cl}}^{-}$ due to the formation of low active radical ${\mathrm{ClOH}}^{•-}$ (Equation (32)) [127].

(30) $${\mathrm{SO}}_{4}^{•-}+{\mathrm{Cl}}^{-}\;\to {\mathrm{SO}}_{4}^{2-}+{\mathrm{Cl}}^{•}$$

(31) $${\mathrm{Cl}}^{•}\;+{\mathrm{Cl}}^{-}\;\to {\mathrm{Cl}}_{2}^{•-}$$

(32) $${\mathrm{HO}}^{•}+{\mathrm{Cl}}^{-}\;\to {\mathrm{ClOH}}^{•-}$$

Nitrate (${\mathrm{NO}}_{3}^{-}$) and nitrite (${\mathrm{NO}}_{2}^{-}$) can be commonly found in various water matrices [119]. Both ${\mathrm{NO}}_{3}^{-}$ and ${\mathrm{NO}}_{2}^{-}$ are able to react with ${\mathrm{SO}}_{4}^{•-}$ to generate low reactive ${\mathrm{NO}}_{3}^{•}$ (E_0_ = 2.3–2.5 V) and ${\mathrm{NO}}_{2}^{•}$ radicals (E_0_ = 1.03 V) (Equations (33)–(34)) [25]. The reaction rate of ${\mathrm{SO}}_{4}^{•-}$ with ${\mathrm{NO}}_{3}^{-}$ and ${\mathrm{NO}}_{2}^{-}$ are 5 × 10^4^ M^−1^s^−1^ and 8.8 × 10^8^ M^−1^s^−1^, separately [45]. Thus, ${\mathrm{NO}}_{2}^{-}$, compared with ${\mathrm{NO}}_{3}^{-}$, has higher reactivity in ${\mathrm{SO}}_{4}^{•-}$ consumption. In addition and in a similar way, ${\mathrm{NO}}_{2}^{-}$ was also reported as the sink of ${\mathrm{HO}}^{•}$ radicals (Equation (35)) [128].

(33) $${\mathrm{SO}}_{4}^{•-}+{\mathrm{NO}}_{3}^{-}\;\to {\mathrm{SO}}_{4}^{2-}+{\mathrm{NO}}_{3}^{•}$$

(34) $${\mathrm{SO}}_{4}^{•-}+{\mathrm{NO}}_{2}^{-}\;\to {\mathrm{SO}}_{4}^{2-}+{\mathrm{NO}}_{2}^{•}$$

(35) $${\mathrm{HO}}^{•}+{\mathrm{NO}}_{2}^{-}\;\to {\mathrm{OH}}^{-}+{\mathrm{NO}}_{2}^{•}$$

## 5. Summary and Outlooks

This review summarized the activation of PMS and PDS by manganese(III) (oxyhydr)oxides for the degradation of recalcitrant pollutants. The desirable morphologies and facets (e.g., cubic structure with (001) facet exposure) can effectively enhance the reactivity of Mn(III) (oxyhydr)oxides in the activation of PDS and PMS. Mn(III) (oxyhydr)oxides showed different reactivity in radical precursors activation. Specifically, both radical (for example, sulfate and hydroxyl radical) and non-radical (such as singlet oxygen) were generated in the Mn(III) (oxyhydr)oxide-activated PMS system. The activation of PDS by α-Mn_2_O_3_ and γ-MnOOH were mainly through the formation of singlet oxygen and the catalyst surface activated complex. The activity of Mn(III) (oxyhydr)oxides in PDS and PMS activation can be influenced by the solution pH due to the occurrence of the electrostatic effect. Moreover, the inhibition effect of inorganic anions (such as carbonate/bicarbonate ions, chloride ions, and nitrate/nitrite ions) on the catalytic performance of Mn(III) (oxyhydr)oxides were discussed in detail. 

Given this comprehensive summary, some future outlooks are proposed. 

Although previous studies already identified the generation of ^1^O_2_ in α-Mn_2_O_3_/PDS system using the ESR and quenching experiments, the detailed catalytic process of PDS on the surface of Mn_2_O_3_ remains elusive. Further studies are needed for a better understanding of the activation mechanism of PDS by α-Mn_2_O_3_. Second, detailed studies are required to exploit the potential application of α-Mn_2_O_3_ or γ-MnOOH-based composites in PDS activation to understand the synergistic performance of α-Mn_2_O_3_ or γ-MnOOH with other loaded materials (such as active carbon, graphene, and transition metals). This will open up new research avenues in the field of water remediation technologies, with the aim to improve the reactivity of α-Mn_2_O_3_/γ-MnOOH in PDS activation. 

The high-scale or industrial application of SR-AOP seems difficult to implement, and that merits being resolved. The development of new modeling approaches that account for the upscaling of different involved reactions and the complexity of heterogeneous reactions at Mn-oxides/water interfaces becomes urgent. More experimental work is also needed to develop new Mn-bearing oxides supported with high catalytic efficiency, suitable for industrial applications, and yet are relevant from both economic and environmental points of view.

## Figures and Tables

**Table 1:** The structures of common Mn(III) (oxyhydr)oxides [50,51].

Mineral Name	Chemical Formula	Mn Valence	Crystal Structure
Mn(III) oxide	α-Mn_2_O_3_	III	Bixbyite
Groutite	α-MnOOH	III	Tunnel
Feitknechtite	ꞵ-MnOOH	III	Layer
Manganite	γ-MnOOH	III	Tunnel
Hausmannite	Mn_3_O_4_	II/III	Spinel

## Data Availability

Not applicable.
